# An evidence map of actigraphy studies exploring longitudinal associations between rest-activity rhythms and course and outcome of bipolar disorders

**DOI:** 10.1186/s40345-020-00200-6

**Published:** 2020-12-01

**Authors:** Jan Scott, Francesc Colom, Allan Young, Frank Bellivier, Bruno Etain

**Affiliations:** 1 Institute of Neuroscience, Newcastle University, Newcastle upon Tyne, UK; 2 Centre for Affective Disorders, Department of Psychological Medicine, Institute of Psychiatry, Psychology & Neuroscience, King’s College London, London, UK; 3 Université Paris Diderot, Sorbonne Paris Cité, UMR-S 1144, 75013 Paris, France; 4 IMIM-Hospital del Mar-CIBERSAM, Barcelona, Catalonia Spain; 5 Universitat Autònoma de Barcelona Barcelona-Catalonia, Barcelona, Spain; 6 Département de Psychiatrie Et de Médecine Addictologique, AP-HP, GH Saint-Louis – Lariboisière – F. Widal, 75475 Paris, France; 7 Inserm U114475006, Paris, France; 8 South London and Maudsley NHS Foundation Trust, Bethlem Royal Hospital, Kent, UK

**Keywords:** Evidence map, Actigraphy, Longitudinal, Bipolar, Response, Course, Modifiers, Lithium, Phenotype, Domains

## Abstract

**Background:**

Evidence mapping is a structured approach used to synthesize the state-of-the-art in an emerging field of research when systematic reviews or meta-analyses are deemed inappropriate. We employed this strategy to summarise knowledge regarding longitudinal ecological monitoring of rest-activity rhythms (RAR) and disease modifiers, course of illness, treatment response or outcome in bipolar disorders (BD).

**Structure:**

We had two key aims: (1) to determine the number and type of actigraphy studies of in BD that explored data regarding: outcome over time (e.g. relapse/recurrence according to polarity, or recovery/remission), treatment response or illness trajectories and (2) to examine the range of actigraphy metrics that can be used to estimate disruptions of RAR and describe which individual circadian rhythm or sleep–wake cycle parameters are most consistently associated with outcome over time in BD. The mapping process incorporated four steps: clarifying the project focus, describing boundaries and ‘coordinates’ for mapping, searching the literature and producing a brief synopsis with summary charts of the key outputs. Twenty-seven independent studies (reported in 29 publications) were eligible for inclusion in the map. Most were small-scale, with the median sample size being 15 per study and median duration of actigraphy being about 7 days (range 1–210). Interestingly, 17 studies comprised wholly or partly of inpatients (63%). The available evidence indicated that a discrete number of RAR metrics are more consistently associated with transition between different phases of BD and/or may be predictive of longitudinal course of illness or treatment response. The metrics that show the most frequent associations represent markers of the amount, timing, or variability of RAR rather than the sleep quality metrics that are frequently targeted in contemporary studies of BD.

**Conclusions:**

Despite 50 years of research, use of actigraphy to assess RAR in longitudinal studies and examination of these metrics and treatment response, course and outcome of BD is under-investigated. This is in marked contrast to the extensive literature on case–control or cross-sectional studies of actigraphy, especially typical sleep analysis metrics in BD. However, given the encouraging findings on putative RAR markers, we recommend increased study of putative circadian phenotypes of BD.

## Introduction

In the 2017 update on the global burden of disease demonstrates that, the four most burdensome non-communicable conditions (in terms of DALYs: disability adjusted life years) are: cardiovascular disease (CVD), cancers, musculoskeletal conditions, and mental disorders (GBD 2017; Whiteford et al. 2013). In individuals aged 15–49, mental disorders in general, and mood disorders specifically, are regarded as the most burdensome conditions worldwide. In the last 3 to 4 decades, considerable progress has been made in the management of CVD and cancer through precision medicine. The application of precision medicine is in its infancy in psychiatry, but funding initiatives (such as the European H2020 platform) are supporting studies of personalized diagnostics and therapeutics across a range of mental conditions including bipolar disorders (BD) (Schumann et al. 2014). Like precision approaches to other chronic disorders, these studies have largely focused on more quantifiable metrics (such as ‘omics’ and brain scanning) as an attempt to increase the biological validity of observed symptoms (Frey et al. 2013; Hellwig and Domschke 2019; MacDonald et al. 2019). This strategy also reflects the proposals described in the RDOC (research domain criteria) paradigm that identifies key domains and analytic units for translational research (Cuthbert and Insel 2013; Insel 2014). Although personalized diagnostics for BD are likely to remain a long-term aspiration, there is emerging evidence that multi-platform, integrated science approaches could offer a pathway for stratifying individuals with BD to improve outcome prediction, namely treatment response, relapse and recurrence, and individual illness trajectories (Scott et al. 2018). However, as these efforts progress, there is a need to extent the search for phenotypes beyond laboratory settings and to test potential biomarkers in real-world settings, such as field and cohort studies, comparative effectiveness research, and large-scale pragmatic trials (Brietzke et al. 2019; Scott et al. 2019).

One of the domains identified by RDOC is the arousal and regulatory system, and RDOC suggests that sleep–wake cycle and circadian rhythms (which we will refer to as rest-activity rhythms or RAR) represent a core construct for investigation (Insel 2014; Smagula 2016). This is of relevance to BD, as converging evidence indicates that RAR disruptions can differentiate BD cases from healthy controls (HC) and/or other comparator groups (Ritter et al. 2011; Geoffroy et al. 2015; Ng et al. 2015; De Crescenzo et al. 2017; Scott et al. 2017). Further, real-time monitoring of most of these phenomena may be possible with wrist-worn devices. Of course, there has been an exponential increase in the development and use of consumer grade wearables (and smart phone apps) in clinical populations (Faurolt-Jepson et al. 2012; Krane-Gartiser et al. 2019). However, many demonstrate low inter-device reliability, none of the apps or programmes are currently validated against ‘gold standard’ measures and few can accurately estimate key markers of circadian timing (Zee et al. 2014; FDA 2019; Depner et al. 2020). The lack of regulatory approval and absence of basic research on or rigour of testing their performance quality means consumer devices are not currently recommended for use in this research field (Baron 2018; FDA 2019; Moar 2018). Currently, RDOC indicates that actigraphy could be applied as an ‘analytic unit’ for longitudinal studies of circadian rhythms and sleep–wake cycles (Cuthbert 2014). Such studies are now underway, but there is no consensus regarding the most useful actigraphy-derived RAR parameters to estimate and/or report (Scott et al. 2019; Smagula 2016. Also, a preliminary scoping exercise of this topic suggested that relatively few actigraphy studies have employed a longitudinal or prospective design in BD. Therefore, we decided to collate any existing publications on these issues and synthesise the current state of knowledge.

This paper has three aims:

- To determine the number and type of actigraphy studies of samples comprised wholly or partly of individuals with BD that explore data regarding: outcome over time (e.g. relapse/recurrence according to polarity, or recovery/remission), treatment response or illness trajectories.
- To examine the range of actigraphy metrics that can be used to estimate disruptions of RAR and describe which individual circadian rhythm or sleep–wake cycle parameters are most consistently associated with outcome over time in BD.
- To demonstrate the use of evidence mapping as a process for synthesising the state-of-the-art in the field of ecological monitoring of RAR and disease modifiers, course of illness, treatment response or outcome in BD.

## Methods

As the use of evidence-mapping dictates the structure of the paper, we provide a brief overview of this approach in Table 1. Below, we outline the application to the project described in this article.

**Table 1:** Brief overview of evidence mapping

Evidence Mapping Evidence mapping is defined as- *“a form of knowledge synthesis that addresses an exploratory research question aimed at mapping key concepts, types of evidence, and gaps in research related to a defined area or field by systematically searching, selecting, and synthesizing existing understanding”* The gold standard methods used in evidence-based research are systematic review and meta-analysis. These classic approaches are both rigorous and provide readers with detailed information about narrow questions (e.g. the efficacy of drug X for disorder Y). However, developments from this archetypal model are now being developed to meet more diverse needs regarding evidence synthesis (Arksey 2003; Miake-Lye et al. 2016). The obvious example is the use of rapid reviews (which address urgent topics and do not always fully adhere to systematic methodologies) or scoping reviews (that identify extensive bodies of literature but do not usually provide detailed synthesis) (Colquhoun et al. 2014). In this context, evidence mapping has emerged as another process that allows researchers and their audience to develop an understanding of the extent and distribution of evidence in on a broad topic, highlighting what is known and also where gaps exist (Katz et al. 2003; Hetrick et al. 2010). Although it applies systematic and replicable methodology, the process is iterative, as expert consensus and preliminary literature searches inform the next steps in the review, so the final product is broader and less detailed than the traditional approaches (Vallarino et al. 2015). Furthermore, the goal is not to provide detailed statistical comparisons, but rather to offer an essential snapshot of what is or is not known at this moment in time. It is presumed that an evidence map for any given field will be followed up in the future when research develops to a point where it becomes justifiable to apply more formal reviews and pooled analyses The depth of evidence synthesized will differ depending on its purpose of an evidence map e.g. an overview of the extent, range and nature of research activity might entirely exclude any details of study findings (Miake-Lye et al. 2016). However, if the latter are considered, they can be represented by graphs and figures, instead of or as well as a detailed table such as the summary tables provided in systematic reviews (Hetrick et al. 2010) *Given the proposed scope of this map, we determined that a simple written summary with basic tables and figures would be offered in the results section of the paper. A detailed Table summarizing sample characteristics and research findings (in an Appendix) and citations in the reference list only would ensure readers focused on key findings we summarize. Individual readers could then choose to examine supporting evidence and relevant references for themes that they wished to investigate further*	

### Rationale for use of mapping

As noted in the introduction, the goal of this evidence map is to present an overview of the extent, range, nature, and findings of clinical research on RAR metrics measured by actigraphy and a range of potential outcomes of BD. Like many evidence maps, the rationale for using this process was determined by prior knowledge of the field. Many research groups, including our own, have synthesized research findings or undertaken meta-analyses of pooled data regarding BD cases compared with HC or other (comparator) groups (Ritter et al. 2011; Geoffroy et al. 2015). Some of those projects identified small-scale studies that used real-time monitoring of BD cases and/or that examined associations between baseline actigraphy recordings and longitudinal course and outcome of BD (Scott et al. 2017). Nearly all of those projects were undertaken several decades ago and differences in sampling, diagnosis or research methods meant the publications failed to meet eligibility criteria for systematic reviews focused on a specific research question (De Crescenzo et al. 2017). Further, it was known that most of the reported data were insufficient (in terms of amount or quality) to merit inclusion in meta-analyses (Ng et al. 2015). As such, an evidence map offered a realistic option to gain an understanding of existing research in this area, especially capturing insights from multiple small-scale studies and mapping provides a means of summarizing the range of publications and findings in a transparent way (Vallarino et al. 2015). The potential advantage over a basic narrative or selective review is that the search process is reproducible, and the map specifically addresses gaps in the knowledgebase (Hetrick et al. 2010). However, findings are described in brief, so a map does not aim to offer the detailed lists of citations and extensive tables included in systematic reviews or meta-analyses.

### Mapping process

The process usually incorporates four steps (clarifying focus; describing boundaries and ‘coordinates’ for mapping; literature search; charting the output maps). After consultation among the co-authors, the questions and scope of the mapping project were defined as follows-

#### Step 1: Clarification of the focus of the map.

It was agreed that the focus would be:

- What evidence exists on this topic and based on a review of study characteristics and methodologies: what conclusions can be drawn about the quality of evidence?
- What areas are, or are not, well-researched?
- Based on the available evidence, what advice can be offered to clinicians and investigators in the field?

#### Step 2: Description of the eligibility criteria (boundaries for inclusion and exclusion), specification of co-ordinates to be mapped (study characteristics associated with outcome/response, etc.) and definition of key variables (RAR metrics):

(a) Studies were eligible if:

Inclusion criteria-

i) The sample or cohort was wholly or partly comprised of BD cases that met clinical or research diagnostic criteria
ii) It reported associations between any RAR actigraphy metric recorded at baseline and a clinical or research-defined outcome assessed at follow-up in BD cases (or vice versa). Note: For the purposes of the map, the focus was on BD cases, so we extracted information on inter-individual or intra-individual changes in actigraphy or outcomes in the clinical cases (i.e. we aimed to avoid focusing on findings of case–control studies or cross-sectional comparisons of RAR metrics between BD and HC, which have been reviewed many times before).
iii) The publication reported any association between RAR metrics recorded by actigraphy and the outcome or response of BD in the short or long term (this could include e.g. naturalistic observational studies of treatment interventions and/or randomized controlled trials).
iv) Presence of comorbidities was not an exclusion criterion, but details were noted.
v) Findings from self-report questionnaires, consumer grade wearables or smartphone apps were eligible if the study also reported data for actigraphy recordings and/or the RAR findings extracted from questionnaires or wearables were reported alongside the equivalent metric derived from actigraphy.

Exclusion criteria-

The map aimed to capture the extent of the existing literature, so there were minimal exclusion criteria. The most significant criterion was that studies that failed to report any measures (quantity and/or or timing) of daytime activity were ineligible. Also excluded were single case reports or studies where the recruitment of participants was not based on a current diagnosis of BD or depression (UP and BD) e.g. studies of individuals at risk of BD or of individuals attending medical clinics (actigraphy has been used to assess treatment outcome in CVD and cancer).

(b) Study Coordinates.

It was agreed that the following would be mapped: year and geographic location of study (we also noted if the study specified seasonality, etc.); sample characteristics (demographics; diagnoses e.g. BD subtypes, inclusion of UP and BD; phase of illness at recruitment); community or clinical setting (e.g. in- or outpatient); duration of study; duration of actigraphy recording; and outcomes reported. The latter could include: outcome prediction; response to introduction of treatment or treatment withdrawal; acute or long-term treatment effects; longitudinal course of BD (continuous or repeated cross-sectional assessment) in terms of illness progression (naturalist follow-up or associated with treatment introduction or withdrawal), symptom exacerbation, relapse/recurrence/recovery/remission and/or putative disease course modifiers (as defined in a study). Also eligible were: naturalistic observational studies in clinician settings (unspecified/uncontrolled treatment), naturalistic monitoring of illness with unspecified/uncontrolled treatment and/or randomized controlled trials that incorporated actigraphy (if not identified via previous criteria).

(c) Definitions of Rest-Activity Rhythms.

It is clear from actigraphy studies in BD that the metrics reported have differed by location (i.e. geography) and decade of study. However, researchers have repeatedly acknowledged the close connection between sleep and circadian structure and that actigraphy data can be used for ‘rhythmometric analysis’ of RAR (Calogiuri et al. 2013; Smagula, 2016; Wirz-Justice, 2007). As such, all the metrics listed in Table 2 are considered as potentially relevant RAR markers in this project.

**Table 2:** Potential measures of rest-activity rhythms (further details are given in Appendix)

Parametric (Cosinor) Model	Non-parametric Model	Sleep Quantity Analysis Model (Mean or Variability)	Sleep Analysis- Circadian Phase Model
Midline estimating statistic of rhythm (MESOR)	Inter-daily Stability (IS)	Mean Activity (MA)	Sleep Onset
Amplitude	Intra-daily Variability (IV)	Time in Bed (TIB) (± Lights Off)	Sleep Offset
Acrophase	Least active period: L5/ L5 Onset	Total Sleep Time (TST)	Sleep Midpoint
	Most active period:M10/M10 Onset	Sleep Onset Latency (SOL)	Sleep Regularity Index (SRI)
	Amplitude	Sleep Efficiency (SE %)	
	Relative Amplitude	Fragmentation Index (FI %)	
		Wake after sleep onset (WASO) (± N awakenings > 5 min)	

Table 2 gives an overview of the key RAR metrics that have been derived from actigraphy recordings in psychiatry (definitions and descriptions of each metric are provided in Table 3 in Appendix) (Calogiuri et al. 2013; Ancoli-Israel et al. 2003). As shown, investigators have focused on different rhythmometric procedures, which need to be considered when interpreting the map. For instance, early studies were likely to report parametric statistics (especially in the USA) (Nelson et al. 1979). Three variables (mesor, acrophase and amplitude) were estimated using cosinor methods (with the p value signifying the probability that the data really show circadian periodicity), whilst more recently these metrics have been derived using regression techniques (which report similar variables but assume more complex patterns and rhythms and are more robust for larger study populations) (Fernandez and Hermida 1998). Non-parametric methods report a wider range of variables to describe the quantity and timing of activity and rest, and especially provide insights into variability/stability of rhythms and any RAR disruptions (Calogiuri et al. 2013; Natale et al. 2009). They are often preferred to parametric models and it is argued that non-parametric models better represent the complexity of RAR than cosinor models (van Someren et al. 1997). Variables derived from basic sleep analysis (such as total sleep time: TST) are probably the most widely reported measures in research in BD. One reason for this is that these sleep quantity are much easier to estimate from raw data (and do not rely on more complex algorithms). Sleep variables are useful for estimating duration and fragmentation of sleep patterns but are less reflective of circadian rhythmicity. Estimation of variability in values for each sleep parameter is encouraged in contemporary literature on RAR and greater reporting of sleep onset/offset/midpoint or sleep regularity index has been employed to give a greater insight into rhythmometrics (Bei et al. 2016).

#### Step 3: Literature Search

The search approach was the same as utilized in systematic reviews. Search terms were applied to PubMed, MEDLINE, PsycINFO, EMBASE, CINAHL and Web of Science databases No limits were set for language, type of study or date of publication (the original telemetric motion sensors and actometers were used in BD in the early 1970s). The search terms included the full range of RAR metrics and potential outcomes listed in Step 2, supplemented by searches using names of medications (e.g. lithium, carbamazepine) or for research groups known to have contributed publications when this topic initially emerged in the literature. This approach was used to ensure the broadest possible range of publications were identified. Furthermore, hand-searches were undertaken of all reference lists of studies noted in narrative reviews, systematic reviews, and meta- analyses on this or similar topics. The final list of eligible studies was generated based on broad relevance to the scope of this map. Key drivers of selection of publications were that:

- The study reported the use of an actigraphy device to undertake daytime or 24-h monitoring of RAR in BD
- The study endpoint was described in terms of a recognizable clinical outcome or measure of response or change in RAR (if combined with baseline or follow-up clinical measures).

#### Step 4. Charting: screening and positioning the relevant evidence within the map

Based on the specified aims of the study, we agreed three core outputs:

- Nature & quality of research: a Table of study coordinates was planned. Co-authors would review the Table independently and produce a brief written synopsis of the key characteristics of the available research. This information was used to formulate a consensus on the nature and quality of the extant literature.
- Extent of research: two maps would be generated. The first would describe year and country of study to provide a snapshot of research in this field by geography and over time. The second map would give an overview of the most common methodologies and research themes. These would be identified by extracting information regarding study coordinates and coding each of these separately, e.g. setting (e.g. inpatient); type (e.g. naturalistic observational; treatment outcome; response criteria; etc.); reported interventions (e.g. proportion of individuals taking lithium; numbers allocated to psychological intervention); whether interventions were compared (e.g. lithium versus quetiapine). These data could be combined to show the overall pattern of research.
- A map would be generated to identify the range of RAR parameters reported across all studies and which individual metrics appeared to be consistently found to be associated with course and outcome of BD. Such a map can offer a heuristic framework and be the starting point for a systematic review or meta-analysis at a future date.

## Results

Twenty-seven independent studies (reported in 29 publications) were included in the map. Table 4 in Appendix gives an overview of the studies and the core characteristics. To maintain the simplicity expounded in evidence mapping we have not included all the citations after every statement (the citations are in the reference list and the key studies can be easily identified from Table 3).

In the studies in the evidence map, the median age of all participants was 45 years and 60% were female. The median sample size was 15 per study (range: 2–75); 484 individuals with BD (out of a total of 560) participated in actigraphy and the median duration of recordings was 7 days (range 1–210). Studies before 1990 tended to be smaller, but sample size did not appear to be determined by decade of publication, with several small-scale projects or BD subsamples (included in larger samples or cohorts) being followed-up longitudinally in recent studies. Overall, 17 studies comprised wholly or partly of inpatients (63%). Two studies reported daytime activity monitoring only, one study used actigraphy primarily to confirm reported patterns of hypersomnia whilst a RCT examined changes in objective levels of morning activity following a psychological intervention targeted at sleep inertia.

There was evidence that statistical analyses of RAR have become more sophisticated over time, but earlier studies were more likely to address directly the association between RAR and phase of BD (e.g. transitions between mania, and depression), and change in RAR following initiation or withdrawal of treatment (especially lithium) (e.g. Kripke et al. 1979; Wolff et al. 1985). The sampling, design, and analytic strategies of most studies demonstrate several methodological flaws (e.g. repeated testing of small samples with correction, absence of reporting of non-significant outcomes). Overall, the consensus judged the quality of the studies to be modest.

**Fig. 1:**
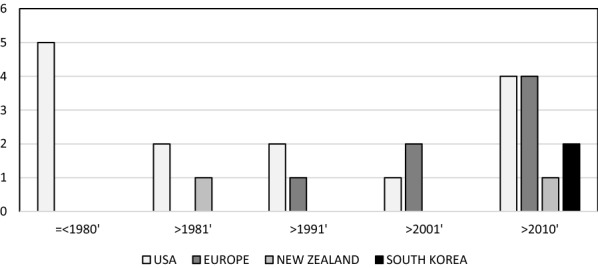
Location of study & decade of publication (Total = 27). (Note: if > 1 publication was identified relating to a dataset from a particular location, we included the date of the first publication only)

Figure 1 shows that > 50% studies (14 of 27) were undertaken in the USA and it is only in the last decade that the number of studies undertaken across Europe has matched the USA. The earliest studies reported the seminal work undertaken by Kupfer and associates and pursued by researchers at NIMH; more recent studies represent projects exploring RAR as disease course modifiers or examining RAR as potential predictors of treatment response and/or to monitor treatment outcomes. Table 3 also demonstrates that there were no studies (meeting our eligibility criteria) that reported RAR in BD for more than a decade (1994–2006). This is most likely a consequence of the fact that psychiatry research shifted towards the reporting of metrics favoured by experts in sleep/sleep disorders, rather than focusing on metrics most relevant to specific psychiatric disorders. Reviewing publications after 2006 suggests this this trend started to reverse when BD researchers increased their focus on sleep–wake cycles and also with the rise in interest in precision diagnostics and therapeutics.

**Fig. 2:**
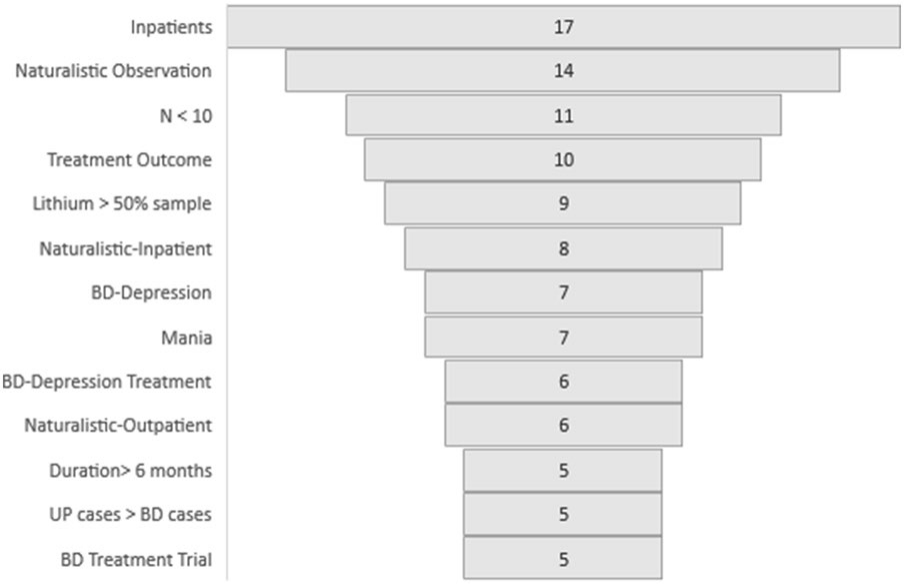
Key coordinates of actigraphy studies – the graph shows the main characteristics or reported purpose of the publications. Note: Only descriptors reported in >  = 5 studies are included in the chart. However, the Total N reported in the graph exceeds the number of citations (n = 28) as most studies are counted in > 1 descriptor category

As shown in Fig. 2, only 22% studies reported follow-up assessments at > 6 months (N = 6). Naturalistic observational studies and treatment outcome studies are equally common (N = 10; 37%). In nine studies, > 50% of the sample were taking lithium or the study specifically explored RAR and lithium treatment.

**Fig. 3:**
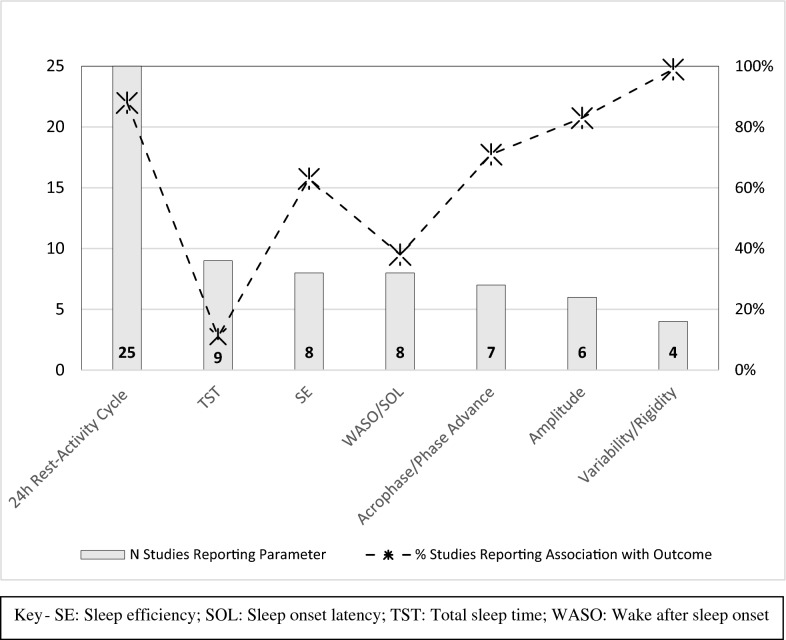
Comparison of number of studies reporting each actigraphy metric & proportion of those studies (%) in which the parameter shows a significant association with an outcome of interest

Although our preliminary work for the evidence-map identified > 30 possible RAR parameters (parametric and non-parametric measures; mean or variable measures of sleep analysis; putative circadian phase markers extracted from sleep quantity analysis), the eligible studies only consistently reported estimates for seven metrics (see Fig. 3). Early studies mainly reported cosinor metrics (amplitude, acrophase and mesor), whilst the more recent studies increasingly report all raw data (either in the main text of the study or Appendices). However, in the middle decades, there was a tendency to only report statistically significant findings, without any indication as to whether other parameters were measured and/or which were found to be non-significant. Interestingly, when number of studies measuring a parameter is compared with the likelihood that a statistically significant association would be found, five variables were associated with outcome in > 50% of the studies. Namely: Variability/Rigidity of RAR (4 out of 4), 24 h rest activity cycle (22 out of 25), amplitude (5 out of 6), acrophase/phase advance (5 out of 7), and sleep efficiency (SE: 5 out of 8). In contrast, most measures of sleep quantity show weaker links: wake after sleep onset and sleep onset latency (WASO/SOL: 3 out of 8) and TST (1 out of 9) being the only metrics showing any significant associations.

It was not possible to provide specific evidence of the nature of the metrics that were most useful in different types of studies, although there was some trend of interest. Using information précised in Table 3, available evidence largely supports the view that mania is associated with circadian phase advance and BD-depression with phase delay. The latter was not obvious in studies of mixed samples of UP and BD depression. Interestingly, treatment may be associated with phase change, and treatments may have different effects on phase in BD-II, and phase shifts may can be associated with outcome at follow-up. The variable 24 h rest-activity cycle represents a global estimate of RAR and its primary value to the map is that it confirms that RAR can be used to assess a range of clinical outcomes in BD. Amplitude appeared to be a useful marker of illness course and treatment response or outcome. It was noted that variability/rigidity of RAR was only estimated in four small samples (Ns = 3, 8, 10, 12). These studies reported extended continuous durations of actigraphy and found the metrics to be significant markers of longitudinal course (including admissions). However, the studies did not report sufficient raw data or basic analyses to allow further investigation or interpretation of the findings.

## Discussion

This article explores associations between RAR metrics measured via actigraphy and course, outcome and/or treatment response in BD. The project arose from three converging strands of work. First, a background scoping exercise undertaken for the H2020-funded consortium exploring a range of putative markers of lithium response phenotypes (including actigraphy) (Scott et al. 2019); second, scrutiny of RDOC publications that suggest undertaking longitudinal actigraphy to explore RAR phenotypes in BD (Insel 2014); and third, a study of circadian and sleep–wake cycles in lithium responders and non-responders (Scott et al. 2020). These projects provided us with sufficient insights into this research field and, alongside our prior knowledge, led us to conclude that the existing literature on RAR and course/outcome/response was unlikely to be suitable for systematic review or meta-analysis. Furthermore, it appeared it would be counter-productive to use these classic evidence-based techniques as they would prematurely restrict the focus of our investigation to a specific or narrow question of what presently seems to be an imprecise research area. As such, it was agreed that utilizing evidence mapping might enable some synthesis of the diverse range of (predominantly small-scale) studies currently available.

Regarding our first question (what evidence exists and what conclusions can be drawn about the quality of evidence?). We conclude that limited evidence exists, but what is available indicates that a discrete number of RAR metrics are more consistently associated with transition between different phases of BD and/or may be predictive of longitudinal course of illness or treatment response. The metrics that show the most frequent associations represent markers of the amount, timing, or variability of RAR rather than the sleep quality metrics that are frequently targeted in contemporary studies of BD. However, these putative ‘circadian’ signals should be viewed in context, as there is evidence of selective reporting of both metrics and outcomes, thus increasing the likelihood of publication bias. Notably, the statistical associations reported derive from multiple small-scale studies that together included < 500 individuals with BD. In sum, the findings are of interest, encourage pursuit of research in this field, but clearly expose that the quality of evidence and available published studies is modest.

Next, the second question addressed what areas are, or are not, well-researched? Our findings about the extent, nature and quality of the research, indicate that the available publications demonstrate the diverse ways in which RAR can be studied in the context of BD, but that no single area has been targeted for a prolonged period of research. Notably, the early studies showed a keen interest in treatment response (or change in illness with treatment withdrawal), but these themes were not pursued to a definitive conclusion. However, this theme is now drawing interest again and two recent studies used actigraphy to explore RAR and acute treatment outcome, namely ketamine infusions for depression (albeit in sample with many more UP than BD cases) and inpatient treatment of mania (such as Blue Blocking Glasses) (Duncan et al. 2017; Henriksen et al. 2020). The map also highlights evidence that clinical depressive symptoms are associated with robustness of circadian rhythm (e.g. Hwang et al. 2017), whilst current RAR patterns may predict future mental state (Salvatore et al. 2008). Also, in a small inpatient sample, it was shown that RAR recorded by actigraphy were associated with clinical progress, whilst estimates made with a consumer wearable were not (Averill et al. 2019). This is a useful reminder that research grade devices are still more accurate than consumer grade device and apps (and so actigraphy is likely to continue to have a role in the immediate future). Importantly, individuals with BD are prepared to wear actiwatches and/or consumer devices for extended periods (> 6 months) and this, allied with new conceptualizations of and analytic approaches to of RAR (such as fractal activity as an indicator system adaptability and lagged data analyses, etc.), may lead to a greater understanding of the chronological sequence of change of RAR, mood, cognition and other cardinal features of BD (Knapen et al. 2020; Walker et al. 2020).

The final question asked, based on the available evidence, what are the potential ways forward for the field? We suggest that one important option would be to develop a consensus within research consortia or wider groups of clinicians and investigators regarding the most appropriate RAR metrics to include in future actigraphy studies of BD (Scott et al. 2017). It is particularly important that this dialogue focuses on metrics that offer insights into RAR in BD that will have the optimal clinical as well as research utility for this field. This should be prioritized over simply generating a list of RAR measures recommended for estimation or reporting of sleep or sleep–wake research in general (Sack et al. 2007). These discussions will necessarily consider any RAR metrics that may represent trait or state variables (e.g. there is emerging evidence that relative amplitude, amplitude, interdaily stability and intradaily variability may be markers of disease onset as well as disease progression) (Merikangas et al. 2019; Scott et al. 2020). Developing a consensus is perhaps the most important recommendation that arises from this mapping exercise, as without such a course of action there is a clear possibility that we will continue to lack the means to make detailed cross-study comparisons of the metrics that would offer most promise as diagnostic or response biomarkers.

An obvious gap in the available literature is the lack of youth studies of actigraphy that take a longitudinal perspective. Given the interest in staging models and temperament, research on the transition between subthreshold conditions or at-risk mental states and the onset of full-threshold illness episodes would be welcome. In individuals with established BD, consideration should be given to RAR metrics that might be combined in multi-platform research of precision diagnostics and therapeutics (Scott et al. 2019). For instance, precision studies that focus on prediction of response to mood stabilizers such as lithium, and the identification of biosignatures (rather than single biomarkers) might consider giving priority to reporting RAR markers such as amplitude and variablility (given our mapping findings regarding emerging evidence for their importance). The mapping exercise demonstrated that actigraphy studies are viable in inpatient as well as outpatient settings, as such, wider consideration should be given to monitoring inpatient progress using objective measures such as actigraphy, with the option of continuation to post-discharge settings. Of course, longitudinal monitoring that allows early warning signs of relapse or recurrent is also feasible with this ecological measure (Bellivier et al. 2015; Ritter et al. 2011). The above steps would address some of the explicit aims outlined by RDOC publications but could be valuable for clinicians e.g. they might consider using electronic monitoring or including sleep diaries as part of the clinical assessment of BD. The latter could be scrutinized not only to identify mean sleep duration and timing but also variability between weekdays and weekends or large differences in duration of sleep onset/offset/duration over 2–3 weeks.

Whilst consumer grade wearables and smart phone apps are attractive to many, it is important to remember that they all await regulatory approval for use as clinical devices (and commercial developments of longer battery life to avoid repeated need for charging). However, their use alongside actigraphy could be helpful, both to validate different measures of RAR that might be derived from consumer devices but also to facilitate ecological momentary assessment (EMA) research (Merikangas et al. 2019; Depner et al. 2020). Research in this area is ongoing and has the advantage of combining self-report ratings of events, cognition, energy, and mood states with objective RAR data (de Wild-Hartmann et al. 2013; Merikangas et al. 2019). However, these advances necessarily require researchers to work more closely alongside statisticians and computational data scientists as the optimal methods for analyzing and understanding links between RAR, symptoms and behaviours are more sophisticated (including non-linear dynamics, entropy) than the basic approaches that are most commonly used and/or the algorithms that are currently available. Assuming this dialogue can occur, combining these approaches would allow to comprehensive exploration of the course of illness, prediction of treatment response and a range of patient reported outcomes (Moskowitz and Young, 2006; Marino et al. 2013). For clinicians, actigraphy offers access to objective ecological monitoring that provides a far more reliable and valid measure of RAR than can be obtained by self-rating scales (Depner et al. 2020; Mulligan 2016). However, not all clinicians feel confident in extracting data and metrics from the devices and this is likely to mitigate against wider dissemination beyond specialist settings. Another advantage of actigraphy is that it is a more feasible option for routine clinical practice than putative ‘omic’ and brain scanning markers of treatment response or illness outcome as these are less available, more expensive and often concentrated in research settings (Gooley and Chua 2014). Also, if the focus is on RAR, then actigraphic monitoring would be easier to integrate into a clinical management and monitoring package than evaluation of other objective circadian markers e.g. measurement of dim light melatonin onset (DLMO) (Depner et al. 2020).

## Conclusions

Despite 50 years of research, and several papers identified by this evidence map being highly cited, use of actigraphy to assess RAR in longitudinal research and examination of these metrics and treatment response, course and outcome of BD is under-investigated. This is in marked contrast to the extensive literature on case–control or cross-sectional studies of actigraphy, especially typical sleep analysis metrics in BD, which are reported in independent studies, systematic reviews, meta-analyses and meta-regressions. The most obvious reason for this marked disparity in the amount of research undertaken using these different methodologies, is that case–control and cross-sectional studies are easier to undertake, usually involve much briefer time frames, and have lower resource requirements than longitudinal research. Also, it was notable that prospective studies identified in this evidence map were still predominantly shorter-term projects, often reporting outcomes over weeks rather than months.

This evidence map suggests that the existence of several often-quoted publications has probably led to an overestimation of the extent and robustness of existing evidence of longitudinal associations between RAR and treatment response, course, and outcome in BD. However, we are aware of promising emerging findings in this area (McCarthy et al. 2019; Scott et al. 2020) and there is tremendous potential for expanding this field of research to inform precision medicine projects in psychiatry. Further, there is a strong argument that, rather than actigraphy being replaced by consumer grade devices and apps, it may be more fruitful to establish frameworks for clinical and research projects that combine these approaches.

## Data Availability

Data sharing is not applicable to this article as no datasets were generated or analysed during the current study. All the data referred to in this manuscript are publicly available in the manuscripts describing the original studies.

## Appendix

See Tables 3 and 4.

**Table 3:** Parameters reported in studies of Rest–Activity Rhythms: additional details

Cosinor model (parametric)	
Midline estimating statistic of rhythm (MESOR)	Estimates the arithmetic mean of the values throughout the 24-h period
Amplitude	Distance from the rhythmic mean to the peak or trough of a mathematical model
Acrophase	Time interval during which the highest values are expected
Nonparametric approach	
Interdaily stability	Quantifies the degree of resemblance between the activity patterns on individual days; ranges from 0 to 1 and may typically be about 0.6 for healthy adults
Intradaily Variability	Quantifies the fragmentation of periods of rest and activity; ranges from 0 to 2 and typically is < 1 for healthy adults, with higher values indicating a more fragmented rhythm
L5	Average of the activity values for the 5 least active consecutive hours in the 24-h cycle
L5 Onset	Onset time of the five most restful consecutive hours
M10	Average of the activity values for the 10 most active consecutive hours in the 24-h cycle
M10 Onset	Onset time of the 10 most active consecutive hours
Amplitude	Difference between M10 and L5
Relative Amplitude	Calculated by dividing AMP by the sum of L5 and M10; ranges from 0 to 1, with higher values indicating higher amplitude of the rhythm
Sleep quantity analysis (reported as Individual Mean values or Intra-Individual Variability e.g. SD, RMSSD)	
Time in bed (TIB) (Lights Off (LO))	Difference between the get-up time and bedtime (Lights Off: time bedroom lights are turned off is reported in some therapy studies)
Total sleep time (TST)	Duration of the sleep episode, calculated on the actual time of sleep start and sleep end
Sleep latency (SOL)	Difference between time in bed and the actual time the monitored subject starts sleeping
Mean activity (MA)	Number of movements within specified epoch (usually 1 min)
Number of awakenings longer than 5 min (NA > 5)	Number of awakenings longer than 5 min
Wake after sleep onset (WASO)	Wake time (min) after sleep onset
Sleep efficiency (SE %)	Percentage of effective sleep during time in bed
Fragmentation Index (FI %)	% of sleep continuity/discontinuity. It is calculated as the amount of time associated with movement (restlessness) during the sleep period expressed as a percentage
Putative Circadian Phase Measures (derived from Sleep quantity analysis data)	
Sleep Onset	Time is the clock time (HH:MM) of the first epoch scored as sleep in each main rest interval
Sleep Offset	Mean sleep offset time is the clock time (HH:MM) of the last epoch scored as sleep in each main rest interval
Sleep midpoint	Calculated as the midpoint between sleep onset and sleep offset
Sleep Regularity Index (SRI)	This is defined as the percentage probability of a person being asleep (or awake) at any two time points 24 h apart

Cosinor model descriptions from Nelson et al (1979); nonparametric approach descriptions from Van Someren et al (1997). Sleep quantity analysis variables and descriptions as from Natale et al (2009), Krane-Gartiser et al. (2019) and Bei et al (2016)

IS quantifies regularity in activity (overall variability in a time-signal after averaging across days) & IV quantifies fragmentation; some argue that SRI best quantifies how rapidly sleep patterns change between consecutive days

**Table 4:** Studies of actigraphic recordings of treatment outcome or course of illness in samples that include individuals diagnosed with bipolar disorders (BD)

Study: 1st author, location, year	Sample characteristics			Actigraphy: number of days (d)^a^	Findings and comments
	N (BD)	Total N*, Diagnoses	Mean Age (SD) yrs; % Female		
Kupfer, USA (1974)	4	N = 11; Bipolar Depression (N = 4) & Unipolar Depression (n = 7)	~ 52 ^b^; 80%	3d at baseline & 3d at wk 3	Baseline (pre-treatment) mean levels of daytime motor activity in un-medicated unipolar depression > BD depression, with significant differences between 19:00 & mid-day (but not mid-day until 19:00) BD cases treated with lithium; UP cases treated with amitriptyline Post-treatment activity differences were no longer evident; (response in Unipolar Depression showed the greatest improvement in activity)
Weiss, USA (1974)	6	Mania (N = 3) & Bipolar Agitated Depression (N = 3)	~ 38 ^b^; 66%	7-9d; additional recording at 20 wks	3 manic inpatients were assessed pre-treatment; then all 6 inpatients were assessed before & after initiation of Lithium. Some individuals repeated actigraphy recordings at 5 months Baseline (pre-treatment) levels of motor activity (counts per minute) in un-medicated mania > agitated depressed, with the most significant differences reported between 22:00 and 02:00 h
Heninger, USA (1977)	13	N = 15; BD-I = 11; BD-II = 2; Unipolar Disorder = 2	55^b^ 84%	2d pre-lithium & 2d at 2 wks	All participants were inpatients who initiated lithium treatment Duration of sleep increased by > 60 min after starting lithium (~ 6.3 h to 7.5 h). When data were reviewed for mania and depression separately, individuals with mania showed reduction in motor activity in parallel with symptom reduction, whilst individuals with depression showed increased motor activity with reduction in symptoms
Kripke, USA (1978)	7	BD: across episodes	28% ^b^	3d (repeated)	Adults assessed through complete cycles of mania and depression. In 5 individuals there was evidence that a circadian rhythm ‘free-ran’ fast & in 5 individuals, evidence suggested that lithium slowed a circadian rhythm. The authors concluded that the prophylactic benefit of lithium may derive from slowing or delaying an overfast circadian clock to prevent desynchronization
Wehr, USA (1980)#	10	Mania & Bipolar Depression	~ 40^b^	2d	Motor activity levels were significantly different across 24 h and indicated ‘phase advance’ of 1–3 h in mania or depression (compared with 14 HC) Acrophase for motor activity was significantly earlier in Mania (13:37 h) & Depression (14:10 h) compared to HC (15:57 h) Acrophase did not differ in 8 BD cases that were assessed in both the Manic & Depressive phases of illness
Godfrey, New Zealand (1984)	4	N = 9; Unipolar Disorder = 5; BD-II, Hypomania = 4	77%^b^	Median ~ 12d	Inpatients wore an actiwatch during the daytime only for 7-27d of the admission. Data were also collected on 9 HC. Daytime activity level was lowest in Depression and highest in HC. During actigraphy, mean daytime activity level increased in Depression & reduced in Hypomania. Change in activity was not correlated with change in depressed mood (no measures reported for hypomania). However, patients continued to show much lower levels of activity compared with HC. (The study abandoned some speech recordings due to low cooperation by some hypomanic patients)
Wolff, USA (1984)#	30	Mania, Bipolar Depression or Euthymia	42 ± 13; 66%	3d	Intra-individual recordings demonstrated that: Mean amount of motor activity in Mania > Depression with the most significant differences being early & later in the daytime Activity levels did not differ significantly in Mania versus Euthymia Total activity (count/24 h) in 18 HC > Euthymic BD
Joffe, USA (1987)	15	N = 19, BD Depression = 15; Unipolar Disorder = 2; Schizoaffective = 1	43 ± 3; 58%	3d pre- & 3d post-initiation of -carbamazepine	Inpatients were included who began carbamazepine treatment after a placebo washout of > 2 wks; actigraphy was undertaken during washout and ~ 35 days later. 7 individuals (37%) met response criteria. Baseline patterns of motor activity were not significantly different in Responders vs. Non-responders Mean 24 h activity increased over time (& mean depression score decreased), with a trend toward greater change in Responder. When time of day was considered, Responders showed higher activity from 13:00 h onwards, with significantly greater activity during evening & night-time
Klein, USA (1991)	4	BD, in remission	Not reported^b^	3d with lithium; 3d at ~ 3 wks post-lithium withdrawal	Long-term lithium was discontinued, and outpatients were followed for 3 months of placebo treatment and up to 12 months. Two individuals experienced manic relapse with symptoms commencing within 2 wks of discontinuation, 2 remained well. Relapse was characterized by fragmentation of sleep–wake patterns, disorganization of 24 h rest-activity cycles & increased motor activity, especially at night. The latter was accompanied by a marked reduction in SE
Klein, USA (1992)	10	BD, in remission	49 ± 12^b^	3d with lithium; 3d at ~ 3 wks post- lithium withdrawal	As with the previous publication, long-term lithium was discontinued, and outpatients were followed for 3 months of placebo treatment. Seven experienced manic relapse: 4 within 3 weeks and all 7 within 3 months of lithium discontinuation. These individuals had higher baseline levels of motor activity (daytime) even though asymptomatic and showed higher daytime & night-time activity (during follow-up) than individuals who remained well (N = 3)
Raoux, France, (1994)	5	N = 26; Unipolar Depression = 21; BD Depression = 5	44 ± 21; 77%	3d treatment-onset & 3d pre-discharge	Inpatients were assessed at beginning & end of admission for melancholic depression (median stay = 26d). All patients had current or past exposure to benzodiazepines. Diurnal hypoactivity, reduced amplitude & longer TST were found at admission. Overall activity level and amplitude increased by discharge. There was an inverse correlation between baseline activity level & clinical improvement (daytime hypoactivity), with a trend for Responders (N = 21) to a lower baseline activity levels than Non-responders (N = 5). Amplitude was a robust marker of response, but acrophase was difficult to interpret because of large intragroup phase variability (~ 25% showed a phase shift of ~ 1 hr)
Todder (2006) & Baune, Germany (2007)	6	N = 26; Treatment-resistant depression: Unipolar = 21; BD-II = 6	49 ± 13; 58%	2 × 11d with 6d gap	Todder reported 4-week follow-up of patients & 27 HC. Sleep parameters in patients showed significant change in the 1st week of follow-up (increased TST & reduced SOL). Even with depression improvement, many sleep parameters differed from HC Baune reported the 4-week study of anti-depressant & quetiapine. At final follow-up, 16 individuals were classified as Responders. Amount of activity had increased significantly at follow-up (& depression score had decreased)
Benedetti, Italy (2007)	39	BD-I Depression	44 ± 10; 54%	7d	Study examined antidepressant effects of sleep deprivation & light therapy; 14 individuals were receiving lithium (36%) Responders (N = 26) & Non-responders (N = 13) did not differ on actigraphy parameters at baseline (although mesor was higher in Responders). Responders showed increase in daytime activity especially in the morning compared with Non-responders. Total sleep was shorter in Responders. Acrophase was advanced in Responders (of ~ 57 min) but delayed by 5 min in Non-responders
Salvatore, USA (2008)#	36	BD-I: Mania/ Mixed States & Remission	44 ± 10; 81%	3d in episode & 3d in remission	BD-I cases were in and outpatients; 72% were prescribed lithium 36 acutely ill BD-I cases were compared with 32 HC at baseline. Then recordings from the BD-I cases when ill & in remission were compared Manic cases showed phase advance (~ 2 h) compared with HC & lower amplitude (mesor & TST did not differ from HC) Mean daytime activity (counts/minute were higher in HC (67 ± 10) than Mania/Mixed States (59 ± 32; but Mania ~ Remission (60 ± 11) Total sleep was significantly longer in remission (Remission > HC; HC = Mania/Mixed States) Acrophase was significantly earlier in Mania/Mixed States (14:01hrs) < Remission (14:26 h) < HC (16:05 h)
Scott, UK (2011)$	10	N = 18 Unipolar Disorder = 8; BD-I = 10	43 ± 12; 60%	30-200d	Naturalistic longitudinal follow-up of individuals attending a lithium clinic who were invited to wear actiwatch for > 1 month (many continued for > 6 months) Lower mean daytime activity (counts/minute) and variability in sleep pattern/duration (alongside psychological measures such as self-esteem) was associated with shorter time to hospital admission during ~ 15-month follow-up (but admissions according to polarity were not reported)
Kim, (2014) & Hwang (2017) South Korea	29	BD-II = 26; BD Depression	36 ± 10; 55%	56d	Open label trial of lithium v. quetiapine (actual N for each group included in analyses varied slightly between the two publications). Individuals were drug free at baseline Quetiapine group (N = 10–12): SE increased (~ 6%) & WASO decreased at 6 weeks follow-up & SE improvement maintained at final follow-up (8 weeks) Lithium group (N = 15–17): Modest improvement in SE (~ 1.5%), other parameters showed some fluctuation without obvious improvement Both quetiapine & lithium affected several circadian parameters, including peak activity time and robustness of circadian rhythm, but exerted different effects on acrophase; clinical depressive symptoms were associated with robustness of circadian rhythm during the 8-week treatment phase
Novak, Czech Republic (2014)	6	BD-I	~ 37 ^b^; 66%	Median ~ 210d	8 individuals were recruited, 7 agreed to participate and 6 of them provided long-term recordings (5–80 months, but about 30% loss of data). 4 individuals reported no relapses, one reported 3 episodes (over 37 months) & one reported 14 (over 80 months). No manic relapses occurred The best signal for identifying relapse was for IS (inter-daily stability)- a marker of regularity of rest-activity patterns (overall classification ~ 66%)
Kaplan, USA (2015)	75	N = 159, BD- I = 143, BD-II = 13; BD NOS = 3	36 ± 11; 66%	7d	159 individuals with BD spectrum disorders were assessed for hypersomnia & followed-up at 7 months later (222 ± 73d). Baseline actigraphy recordings in 75 individuals identified 2 forms of hypersomnia: ‘long sleepers’ (although actigraphy showed these individuals spent longer in bed but did not have longer TST) and those with excessive sleepiness (lower daytime activity) During follow-up, 21 individuals (17.5%) had a hypomanic/manic & 37 (30.1%) a major depressive episode. Self-reported ‘Excessive daytime sleepiness’ predicted relapse into hypomania/mania (p < 0.01); actigraphy was not used to directly assess objective markers of relapse
Gershon, USA (2016)	37	BD-I	35 ± 10; 62%	42d	Lower amount of daytime activity & later daily onset of activity were both associated with greater likelihood of onset of a depressive episode &/or increase in depressive symptom severity. Weaker evidence suggested a slightly elevated mid-day peak & less evening activity were also noted
Moon, South Korea (2016)	26	BD-I = 23 Manic = 21; Depressed = 4. (Manic & Depressive Episodes = 1)	30 ± 11; 62%	At admission; 2 wks, 4 wks & recovery	All BD cases were recruited during a hospital admission. Analyses included 26 manic and 5 depressive episodes. Final wake time was delayed by ~ 1 h and SE improved by ~ 4% during admission Advanced phase was recorded in mania but returned to normal after treatment; likewise, delayed phase in depression returned to normal after treatment. These shifts led to BD cases more closely resembling HC (N = 18) Phase advance or delay observed in biochemical rhythms (such as cortisol) were much greater than sleep–wake cycle changes identified by actigraphy
Bewernick, Germany (2017)	2	N = 16; Unipolar Depression = 14; BD Depression = 2	49 ± 9; 66%	7d	12 individuals (10 inpatients) completed the study. Actigraphy was not undertaken during sleep. Individuals with higher activity levels at baseline showed a larger reduction in symptoms of depression after one week. Change in severity of depression correlated with increased in amount of daytime activity (with significance for some but not all ratings). Activity also showed a lagged correlation with mood rating in the following hours (but not vice versa)
Duncan, USA (2017)	21	N = 51; Bipolar Depression = 21; Unipolar Depression = 30	~ 42; 59%	9d: (4-days pre- & 5 days post-ketamine	Data from 51 trial participants receiving ketamine (21 responders; 30 non-responders) were compared with data from 38 who received placebo Pre-ketamine infusion: evidence of phase advanced activity pattern (acrophase) & lower mesor (144 vs. 167) was associated with good response. Persistent response (day 1 & 3 post-infusion) was characterized by higher amplitude & increased mesor
Krane-Gartiser, Norway (2018)	3	Series of BD-I cases with > = 2 admissions (over 1–6 months)	~ 50^b^	1d per admission	Intra-individual 24-h activity patterns for recurrences of the same polarity show similar patterns; but episodes with different polarity show intra-individual differences in activity patterns. Acute phases were characterized by greater variation between rest-activity and periods with distinct irregularity in activity (measured by sample entropy) in all cases
Averill, New Zealand (2018)	2	N = 24; 22 Unipolar Depression; 2 BD Depression	38 ± 14; 37%	17d	Inpatients were recruited to a study of activity, psychomotor speed & treatment response; usable actigraphy data available from 16 individuals (2 receiving lithium) Change in 24-h activity count was correlated with treatment response Data from consumer grade activity monitor did not correlate with outcomes
Kaplan, USA (2018)	40	Inter-episode BD I (with insomnia)	39 ± 14; 70%	14d (7d pre- & 7d post-intervention)	58 patients were randomized to a two-arm trial; 40 participated in a one session intervention experiment actigraphy data were available on 29 A behavioural intervention (Rise-Up: aimed at overcoming sleep inertia) was compared with a control condition (education: PE) Individuals in the Rise-Up group were significantly more active in the 1^st^ hour upon waking compared to PE
Henriksen, Norway (2020)	20	BD-I, Mania	45 ± 13; 70%	2d (baseline & day 5)	The trail recruited 32 individuals: this study reports data for 20; 10 inpatients were allocated to the blue blocking glasses (BBG) & 10 to placebo glasses 60% of all cases were receiving lithium At day 5: SE improved more in the BBG compared with the control group (4% v 0%). The BBG group showed lower activity count during the sleep period (L5) & lower WASO & a trend for lower FI
Knapen, Netherlands (2020)	14	BD-I	45 ± 12; 79%	~ 180d	Continuous monitoring of temporal relationship between sleep, daily rhythm (measured by actigraphy) and mood changes. Time-lagged association showed significant negative correlation between depression and fractal patterns of motor activity (a measure of rigidity or adaptability of physiological systems) with higher level of depression associated with greater rigidity (with 5–7-day delay)

%, Mean and SD: percentages, means and standard deviations are reported to the nearest whole number; *BD* Bipolar Disorder, *BD-I/II/NOS* Bipolar I/II Disorder, *BD* Not Otherwise Specified, *d* day, *h* hours, *HC* Healthy Controls, *N* number, *NA* not available, *SD* Standard Deviation, *SE* Sleep Efficiency, *SOL* Sleep Onset Latency, *TST* Total Sleep Time, *yrs* years. *Total N is reported only if sample contains cases with other diagnoses (as well as BD); ^a^ Estimated for some studies that did not specify exact time frames; ^b^This either indicates that data were reported as medians and/or data were missing for some variables; ^#^The same sample of BD cases was studied in different mental states; ^$^

Analysis reported by Scott (2011) is described in Scott et al. (2017)

## Abbreviations

BD: Bipolar disorders
CVD: Cardiovascular disease
d: Days
DALYs: Disability adjusted life years
DLMO: Dim light melatonin onset
EMA: Ecological momentary assessment
HC: Healthy controls
IS: Inter-daily stability
IV: Intra-daily variability
Li: Lithium
N: Number
NIMH: National Institute of Mental Health
RDOC: Research domain criteria
RAR: Rest activity rhythms
SD: Standard deviation
SE: Sleep efficiency
SOL: Sleep onset latency
TST: Total sleep time
UP: Unipolar
WASO: Wake after sleep onset
wks: Weeks
yrs: Years
